# Lysosome quality control in health and neurodegenerative diseases

**DOI:** 10.1186/s11658-024-00633-2

**Published:** 2024-09-05

**Authors:** Veronica Ferrari, Barbara Tedesco, Marta Cozzi, Marta Chierichetti, Elena Casarotto, Paola Pramaggiore, Laura Cornaggia, Ali Mohamed, Guglielmo Patelli, Margherita Piccolella, Riccardo Cristofani, Valeria Crippa, Mariarita Galbiati, Angelo Poletti, Paola Rusmini

**Affiliations:** 1 Dipartimento di Scienze Farmacologiche e Biomolecolari “Rodolfo Paoletti”, Università degli Studi di Milano, Dipartimento Di Eccellenza, 2018-2027 Milan, Italy; 2 Unit of Medical Genetics and Neurogenetics, Fondazione IRCCS Istituto Neurologico Carlo Besta, Milan, Italy

**Keywords:** Lysosome, Galectins, Neurodegeneration, Lysosomal membrane permeabilization, Lysosome quality control

## Abstract

Lysosomes are acidic organelles involved in crucial intracellular functions, including the degradation of organelles and protein, membrane repair, phagocytosis, endocytosis, and nutrient sensing. Given these key roles of lysosomes, maintaining their homeostasis is essential for cell viability. Thus, to preserve lysosome integrity and functionality, cells have developed a complex intracellular system, called lysosome quality control (LQC). Several stressors may affect the integrity of lysosomes, causing Lysosomal membrane permeabilization (LMP), in which membrane rupture results in the leakage of luminal hydrolase enzymes into the cytosol. After sensing the damage, LQC either activates lysosome repair, or induces the degradation of the ruptured lysosomes through autophagy. In addition, LQC stimulates the de novo biogenesis of functional lysosomes and lysosome exocytosis. Alterations in LQC give rise to deleterious consequences for cellular homeostasis. Specifically, the persistence of impaired lysosomes or the malfunctioning of lysosomal processes leads to cellular toxicity and death, thereby contributing to the pathogenesis of different disorders, including neurodegenerative diseases (NDs). Recently, several pieces of evidence have underlined the importance of the role of lysosomes in NDs. In this review, we describe the elements of the LQC system, how they cooperate to maintain lysosome homeostasis, and their implication in the pathogenesis of different NDs.

**Graphical Abstract:**

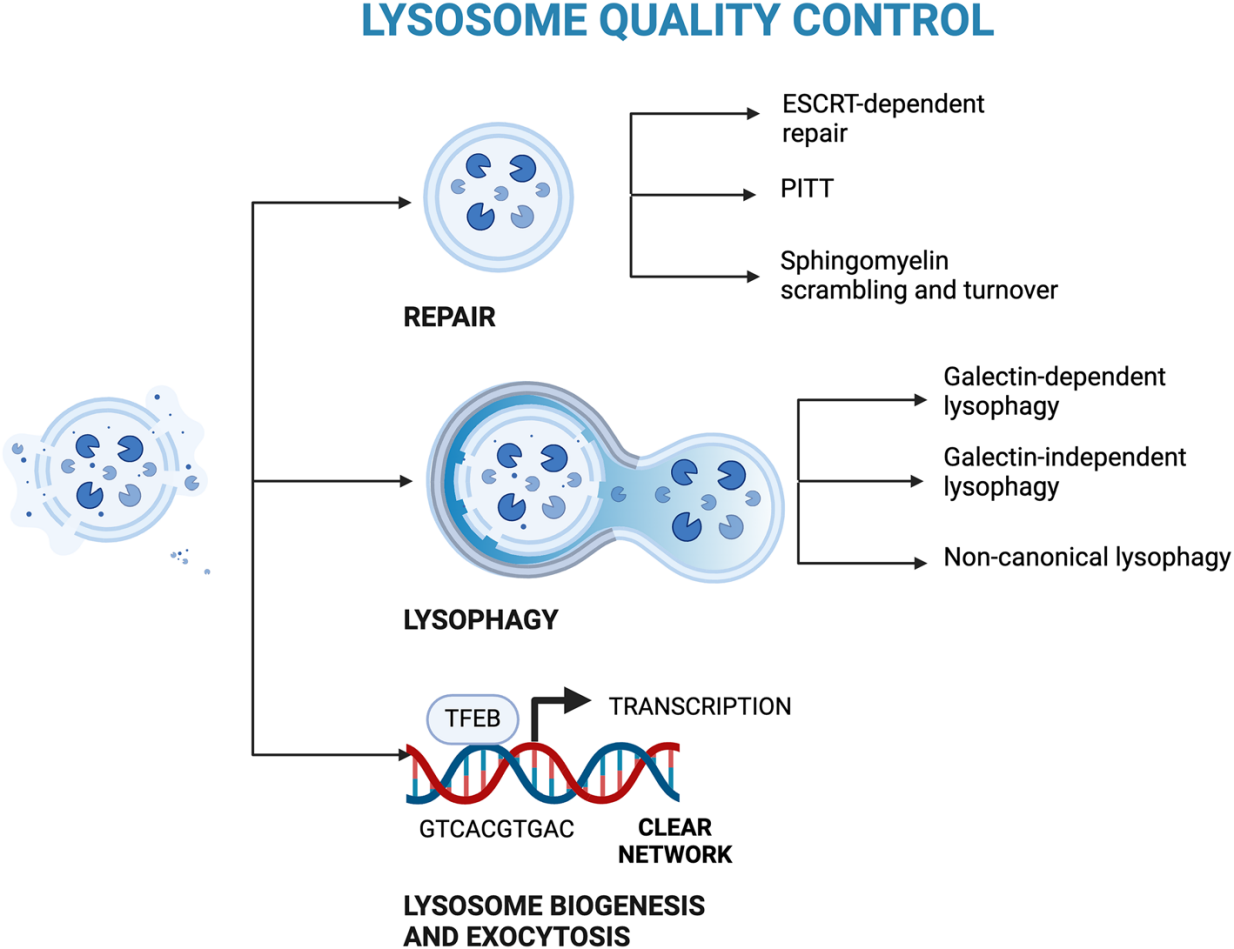

## Lysosomal functions

Lysosomes are membrane-enclosed acidic organelles found in all eukaryotic cells, discovered by De Duve in the middle of the last century [1, 2]. Lysosomes contain a wide range of hydrolases capable of degrading all macromolecules present in cells: nucleic acids, lipids, carbohydrates, proteins, and cell debris. For decades lysosomes have been considered the “trash bin” of cells, without any other specific role [3]. In the last 10 years, however, research on lysosomal function has intensively increased and nowadays these organelles are considered an important hub for cell metabolism and nutrient sensing [4, 5]. Lysosomal functions are involved in: endocytosis, phagocytosis, autophagy [6], lysosome exocytosis and plasma membrane repair [7, 8], control of nutrient sensing [9, 10], and cell death processes [11]. This variety of functions implicates lysosomes in many human diseases. Notably, defects in genes coding for lysosomal enzymes are causative factors in a group of more than 50 inherited metabolic disorders. These disorders, named lysosomal storage disorders (LSDs), are characterized by the lysosomal accumulation of undigested substrates, and include Gaucher disease, Fabry disease, and Neimann–Pick disease [12–14]. Lysosomal dysfunctions have been also found to play important roles in cancer [15, 16] and neurodegenerative diseases (NDs) [17–24]. In most cases, these diseases show adulthood onset and a progressive decline. In addition, the decrease in lysosomal function observed during ageing may contribute to disease pathogenesis [25].

## Lysosomal membrane composition

Extracellular material, intracellular molecules, and organelles are driven to lysosomes for degradation. Lysosomal catabolic functions require that these components are transported and delivered to the lumen of the organelle, where the acidic hydrolytic enzymes degrade the substrates. The integrity of the limiting membrane is crucial for the proper functionality of lysosomes, and this is ensured by a thick membrane (around 8 nm) composed of lipids and glycoproteins with a luminal glycosylated domain [26]. This lysosomal glycocalyx has a protective role in the acidic environment, and it is fundamental for the functionality of the lysosomal membrane proteins [i.e., lysosomal integral membrane proteins (LIMPs) and lysosomal associated membrane proteins (LAMPs)]. Alongside soluble lysosomal hydrolases, lysosomal membrane proteins play a pivotal role in organelle biogenesis and functionality [27, 28]. Among these, LAMP-1, LAMP-2, LIMP-1/CD63 and LIMP-2/SCARB2 are the most abundant, with the former two representing more than 50% of total lysosomal membrane proteins [29]. Since their discovery, LAMP-1 and LAMP-2 have been considered structural molecules committed to ensuring lysosome integrity by protecting the membrane from the acidic luminal compartment [30]. Recently, it has been demonstrated that these proteins perform functions beyond this preservation and that, despite their 37% sequence homology, they show important functional differences from one another. Experiments performed in mice show that the inactivation of the *Lamp1* gene does not alter lysosomal morphology and function [31], while in *Lamp2*-deficient mice, increased cell mortality correlating with the accumulation of autophagic vacuoles (AVs) occurred in several tissues [32]. *Lamp2*-deficient mice model the symptoms of Danon disease in humans, an LSD caused by *Lamp2* mutations characterized by abnormal accumulation of AVs in heart and in skeletal muscle [33, 34].

Another factor that contributes to the stability of the lysosomal membrane is lipid composition. Lysosomal membranes are enriched in sphingomyelin and are characterized by the presence of bis(monoacylglycerol)phosphate, a negatively charged lipid exclusively present in lysosomes [35–37]. Of note, cholesterol is an essential heterogeneously distributed membrane component, mainly present in the plasma membrane [38]; however, the lysosome represents a unique organelle in terms of cholesterol content, since its membrane cholesterol composition is intermediate between that of the plasma membrane and that of other intracellular organelles [39]. Indeed, lysosomes play a key role in maintaining cholesterol homeostasis and in cholesterol dynamics [39–41]. Thus, alterations in the cholesterol content affect the integrity and stability of the lysosomal membrane and a reduction in its levels induces lysosomal membrane permeabilization (LMP; see below for a detailed description) [42]. Conversely, the addition of cholesterol to isolated lysosomes or cell cultures increases lysosomal membrane stabilization [43–45].

## Lysosomal quality control

Given the central role of lysosomes for cellular homeostasis, any stress or alteration that affects lysosomal integrity can critically impact cell viability. To maintain lysosomal functionality, lysosomal damage is recognized and resolved by lysosomal quality control (LQC). The LQC consists of multiple pathways dedicated to lysosomal repair, clearance (lysophagy), exocytosis, and biogenesis. LQC is generally activated in response to LMP, an event characterized by membrane damage, lysosomal swelling, and the release of lysosomal luminal content into the cytosol with possible uncontrolled breakdown of biomolecules [46, 47]. In addition to the previously mentioned changes in cholesterol content in the lysosomal membrane, a variety of exogenous and endogenous factors can cause LMP, including lysosomotropic agents, compounds entrapped in the lysosomes after protonation (l-leucyl-l-leucine methyl ester hydrobromide, glycyl-l-phenylalanine 2-naphthylamide, chloroquine, and the cationic amphiphilic drugs), reactive oxygen species, the apoptotic regulator Bcl-2-like protein 4, and infectious pathogens [48, 49]. LMP may also occur in response to neurotoxic events in NDs (see Lysosomal Damage in Neurodegeneration section for details) [50–56].

### Lysosomal damage recognition and repair

**Fig. 1:**
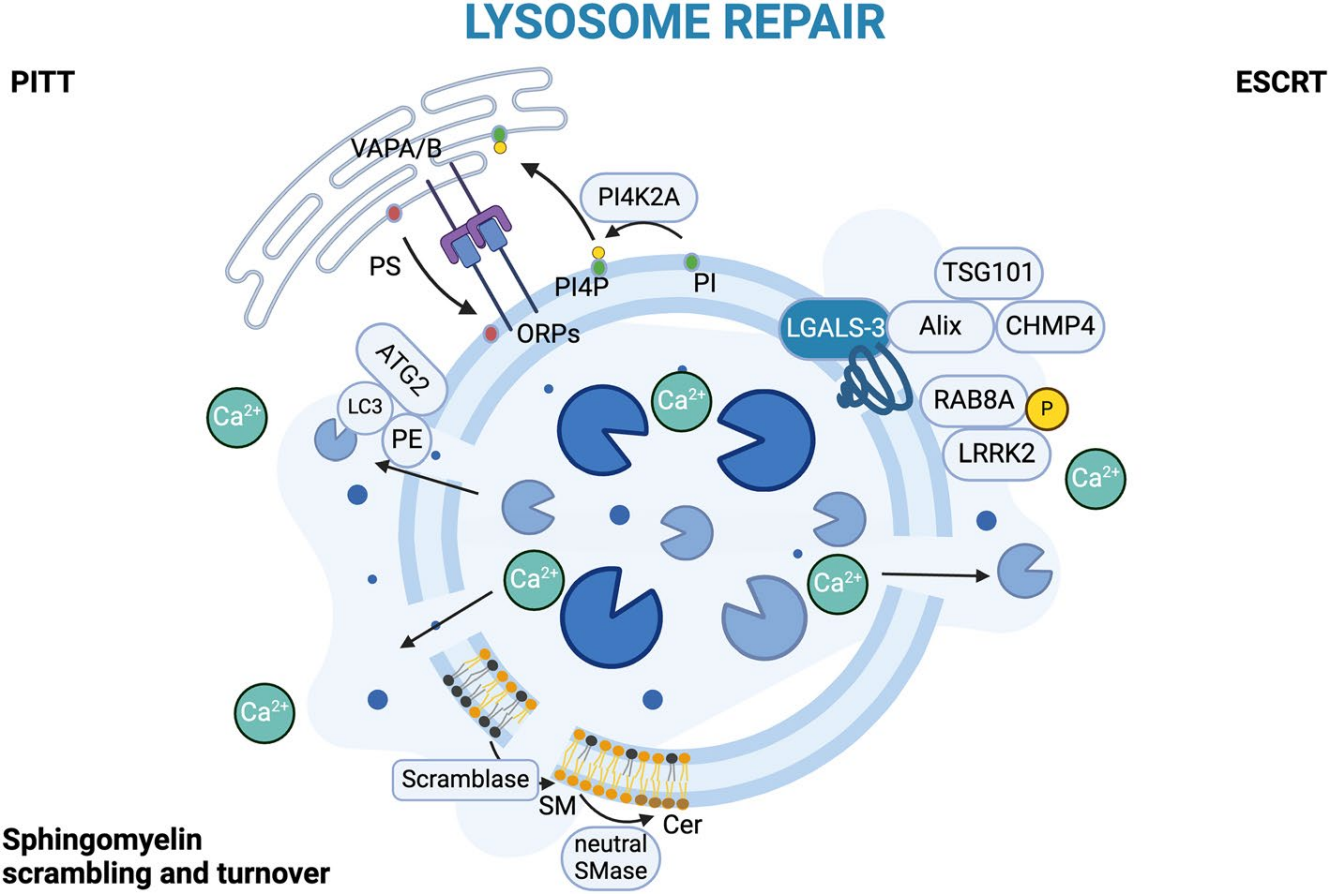
Lysosome repair. Lysosomal membrane permeabilization (LMP) and damage is recognized and repaired by mechanisms such as the PITT, the ESCRT pathways, and the sphingomyelin scrambling and turnover. (i) The PITT mechanisms consist of protein complexes that promote lysosome lipid membrane turnover by interacting with endoplasmic reticulum (ER). (ii) The ESCRT machinery is recruited by galectins and restores lysosome membrane integrity. (iii) The remodeling of damaged lysosomal membrane can be directed by sphingomyelin scrambling and turnover. Created with BioRender.com

To cope with lysosomal damage, cells have sensor proteins capable of recognizing lysosomal damage and activating intracellular responses (Fig. 1). These proteins are the galectins, a group of 15 proteins characterized by the presence of a common carbohydrate recognition domain, a beta-sandwich domain composed of 130–140 residues with high affinity for carbohydrates [57].

Galectins are small, soluble, and dynamic cytosolic proteins, that can shuttle into the nucleus or be secreted into the extracellular environment by unconventional secretion processes [58, 59]. Multiple pathways seem to be implicated in galectin secretion; evidence suggests it can be mediated by direct release, by lysosome/endosome exocytosis, or by extracellular vesicle release [59]. In the extracellular space, secreted galectins may bind to specific beta-galactosides forming a cross-linked complex, a dynamic lattice [60], and play important roles in cell adhesion, cell migration, signaling, immune response, inflammation, and endocytosis [61–64]. At the intracellular level, galectins reside in the cytosol, but, when necessary, they can recognize and bind to damaged endosomal membranes [65, 66], with galectins-3, 8, and 9 being mostly involved. In normal conditions, glyco-conjugates are present in the lumen of lysosomes and endosomes, but upon membrane disruptions, they are exposed to the cytosol and may be recognized by galectins. Indeed, the accumulation of galectins in discrete cytosolic puncta is a hallmark of LMP [67]. In the presence of a small rupture in the lysosomal membrane, galectin-3 translocates to the lysosomes and recruits the programmed cell death 6 interacting protein (PDCD6IP/ALIX), tumor susceptibility 101 (TSG101), the Endosomal sorting complex required for transport III (ESCRT-III) component charged multivesicular body protein 4B (CHMP4B), and VPS4 essential proteins for lysosomal membrane repair [66, 68–70]. This process is followed by lysosomal calcium efflux and triggers the RAB29-mediated translocation of the leucine-rich repeat kinase 2 (LRRK2) to the damaged lysosomes. LRRK2 phosphorylates and engages RAB8A facilitating the recruitment of ESCRT components for membrane repair [17, 71, 72]. Concurrently, LRRK2 also recruits and activates both RAB10 and its protein interactor C-Jun-amino-terminal kinase-Interacting Protein 4 (JIP-4) promoting the lysosomal tubulation sorting, a process driven by LRRK2 and necessary for the release of vesicles from lysosomes [73, 74].

Evidence showing that ESCRT depletion was not fully capable counteracting lysosomal repair suggests that this is not the only lysosomal repair mechanism [68, 70].

Recently, two ESCRT-independent pathways for lysosomal membrane repair have been discovered.

Tan and Finkel identified phosphoinositide-initiated membrane tethering and lipid transport (PITT), a specific set of proteins involved in LMP beyond the known ESCRT components including the membrane-bound phosphatidyl-inositol-4 kinase type 2 alpha (PI4K2A) [75, 76]. This enzyme catalyzes the phosphorylation of phosphatidyl-inositol (PI) to phosphatidyl-inositol 4-phosphate (PI4P), a lipid essential for the endolysosome system, as well as its binding proteins, the oxysterol binding protein (OSBP) related 9, 10, and 11 (ORP9, ORP10 and ORP11). In this alternative repair pathway, LMP induces the activity of PI4K2A leading to the production of PI4P and the lysosomal accumulation of ORP9, ORP10, and ORP11; these are subsequently involved in the formation of inter-organelle membrane contact sites (MCS). ORP proteins dimerize with each other and establish endoplasmic reticulum (ER)-lysosome MCS via the interaction of the ER-resident VAMP-associated proteins A and B, favoring the exchange of PI4P with phosphatidyl-serine (PS) and transporting it to lysosomes. In a complementary way, OSBP transports cholesterol to damaged lysosomes for repair. The accumulation of PS in lysosomes stimulates the non-canonical activity of the autophagy-related 2 (ATG2) protein, involved in lipid transport [76]. The link between the ESCRT-pathway and PITT in lysosomal membrane repair remains to be elucidated.

The second ESCRT-independent mechanism for lysosomal repair has been uncovered by Niekamp and colleagues. The process is triggered by cytosolic exposure of sphingomyelin to the surface of damaged lysosomes catalyzed by the Ca^2+^-dependent scramblase. This is followed by the cleavage of sphingomyelin by neutral sphingomyelinase to produce ceramides facilitating membrane repair [77]. These repair pathways act in parallel to ensure lysosome integrity.

Interestingly, it has been shown that lysosomal damage inactivates mTOR, which normally functions to negatively regulate autophagy and catabolic pathways. This is mediated through a galectin-based system named GALTOR, suggesting a link between lysosomal damage and the regulation of cellular metabolism [78].The system is based on the interaction between galectin-8, the lysosomal aminoacidic transporter solute carrier family 38 member 9,and the ragulator–Rag complex. Concurrently, galectin-9 activates AMP-activated protein kinase (AMPK) increasing its phosphorylating activity via association with the AMPK upstream kinase mitogen-activated protein kinase 7 (MAP3K7/TAK1). The interaction between galectin-9 and the deubiquitinase ubiquitin-specific peptidase 9 X linked (USP9X) governs the lysine 63 ubiquitination (K63) rate of MAP3K7/TAK1, a process that regulates and activates the enzyme. In physiological conditions, USP9X negatively regulates MAP3K7/TAK1 activity by deubiquitination; instead, under LMP conditions, galectin-9 interferes with USP9X promoting MAP3K7/TAK1 ubiquitination and activation [66, 79].

### Lysophagy

**Fig. 2:**
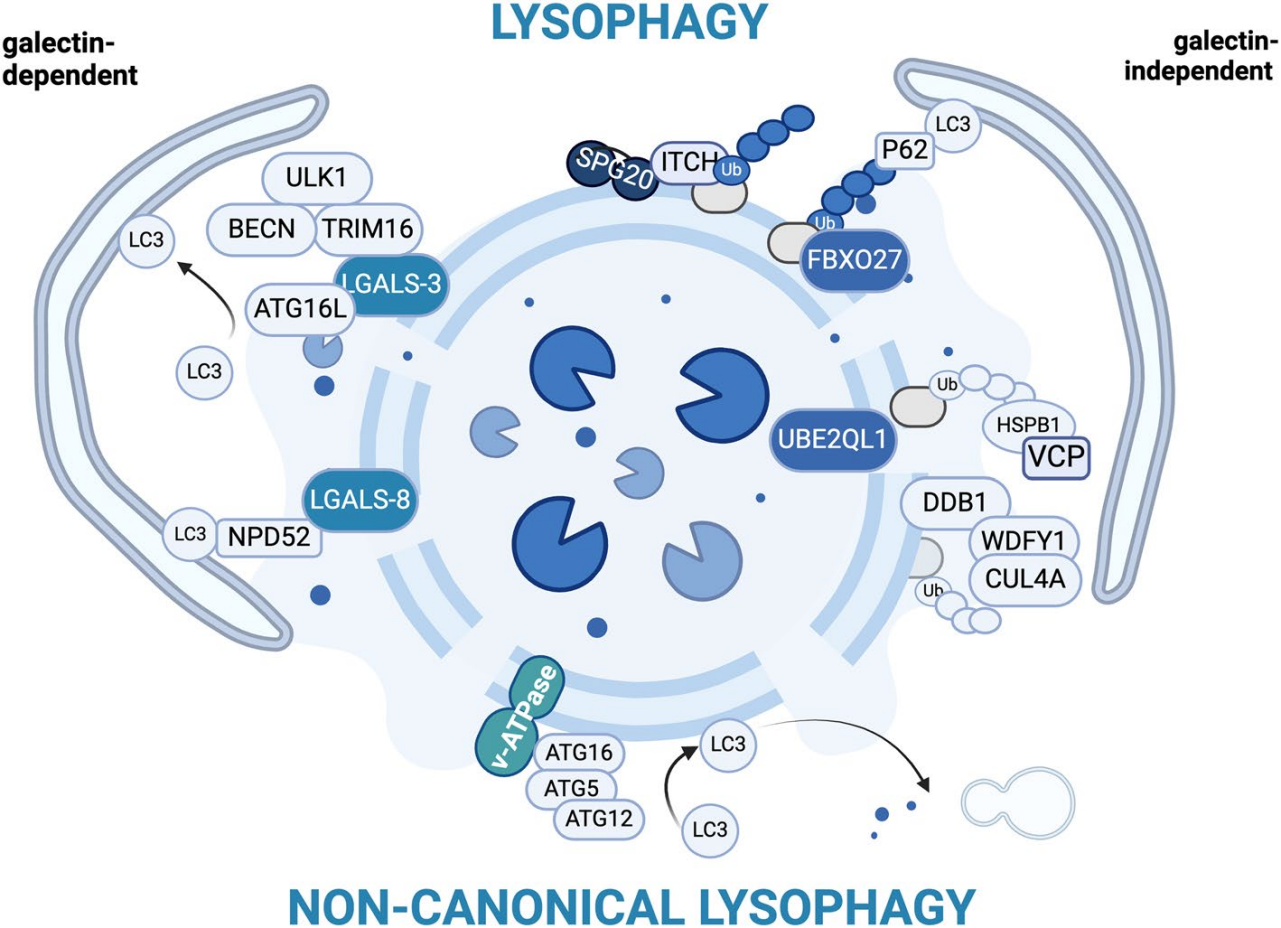
Lysophagy. Lysosome clearance through autophagy is activated when the repair mechanisms fail or the damage persists. Lysosome clearance can occur by marking the damaged lysosomes and recruiting them to the forming phagophore via various galectin-dependent or independent mechanisms. Created with BioRender.com

When the lysosomal membrane cannot be repaired, a complex mechanism is activated to promote the clearance of the whole organelle via selective autophagy, a process known as lysophagy (Fig. 2). Lysophagy is activated when repair mechanisms fail. To date, the exact mechanism responsible for the switch from lysosomal repair to clearance has not been characterized. Indeed, the degradation of damaged lysosomes is mainly triggered by the recruitment of galectins; galectins sense the LMP, bind exposed glycans, and recruit enzymes involved in lysosome ubiquitination (E2, E3 enzymes); these processes mainly involve galectin-3 and galectin-8. Galectin-3 recruits and binds the tripartite motif-containing 16 (TRIM16), an atypical E3 ubiquitin ligase that contributes to lysosome ubiquitination and serves as a platform to recruit autophagy-related proteins such as ULK1, ATG16L, and BECN-1 [52, 80]. These proteins are all part of complexes that together regulate autophagy initiation by mediating the formation of the phagophore, a double-membraned structure, and the activation of microtubule-associated protein 1 light chain 3 (MAP1LC3, or simply LC3). Thus, TRIM16 mediates the interaction between lysosomes and the forming phagophore, facilitating the engulfment of the damaged lysosomes. Moreover, TRIM16 regulates the activation of the transcription factor EB (TFEB), the master regulator of autophagy and lysosome biogenesis (see below for a detailed description) [80]. In parallel to galectin-3, galectin-8 directly binds a specific autophagy receptor (AR), the calcium-binding and coiled-coil domain 2 protein (CALCOCO2, also known as NDP52). NDP52 recruits the forming phagophore and interacts with the ragulator–Rag complex inhibiting mTOR and therefore activating TFEB [66]. Hence, TFEB activation induces the transcription of autophagic genes responsible for lysophagy and lysosomal biogenesis (see below for a detailed description of TFEB activity).

Ubiquitination of damaged lysosomes is also regulated through galectin-independent mechanisms by E2 and E3 enzymes, such as the SKP1/CUL1/F-box (SCF) protein ubiquitin ligase complex, Cullin-4A—DNA damage-binding protein—WD repeat and FYVE domain-containing 1 complex, and UBE2QL1. Ubiquitination can occur on K63- or K48- linked ubiquitin chains. ARs recognize K63-linked ubiquitin conjugates, while K48-linked chains generally are associated with proteasome degradation. K63- and K48-ubiquitinations on lysosomal membrane proteins occur with a different timing and play different roles. K63-ubiquitination arises quickly after the damage, together with the recruitment of the AR sequestosome-1 (SQSTM1/p62). Different ligases can mediate K63 ubiquitination: F-box protein 27, the substrate recognition subunit of the SCF complex, directly binds to glycans and damaged lysosome membranes promoting LAMP-1 and LAMP-2 K63 ubiquitination; ITCH ubiquitylates membrane-associated proteins to initiate lysophagy. ITCH is recruited and activated by SPART/SPG20, a galectin/LMP-independent detector of lipid-packing defects on the lysosome membrane. SPART/SPG20 binds to IST1, a repair factor, and senses membrane defects that precede LMP. When lipid membrane alterations are unacceptable, SPART/SPG20 recruits ITCH initiating autophagy [81, 82].

K48-ubiquitination occurs later on and is mediated by E2 or E3 enzymes such as UBE2QL1 or Cullin-4A [83–85]. The K48 conjugates targeted by UBE2QL1 are recognized by the heat shock protein B1 (HSPB1), which favors their segregation by valosin-containing protein (VCP) and their clearance by the proteasome [86, 87]. The first set of proteins targeted by ubiquitination is ARs, which promote phagophore engulfment [81]. Subsequently, the second set of ubiquitinated proteins is membrane trafficking regulators, such as soluble n-ethylmaleimide-sensitive factor attachment protein receptors, which suppress the fusion of damaged lysosomes with autophagosomes or late endosomes. Finally, the third set of proteins targeted by ubiquitination are proteins that orchestrate the cytoskeleton in lysophagy dynamics, such as cellular communication network factor 2. The clearance of this last set of proteins is regulated by VCP and is necessary for lysosomal degradation [86].

Damaged lysosomes, marked with ubiquitin-chains, are linked to autophagic membranes by ARs [e.g.: SQSTM1/p62, optineurin (OPTN), NDP52, NBR1 autophagy cargo receptor (NBR1), Tax1 binding protein 1]. They possess an ubiquitin-associated domain, which recognizes ubiquitin chains, and an LC3-interacting region, which directly binds LC3 present on the forming phagophore. AR activation is regulated by TANK-binding kinase 1 (TBK1), which by phosphorylating them, increases their affinity to ubiquitin chains [88, 89]. SQSTM1/p62 is the major actor in lysophagy; in fact, it is consistently found on damaged lysosomes and its depletion prevents lysosome clearance [81, 83, 90]. The recruitment of SQSTM1/p62 upon lysosomal damage is regulated by HSPB1. During phagophore formation, HSPB1 is recruited to lysosomes and is phosphorylated to allow its inclusion in the SQSTM1/p62 condensates (also known as p62 bodies) formed by liquid–liquid phase separation (LLPS), favoring the maintenance of its liquid–liquid phase properties, and thus promoting lysophagy [90].

Besides the canonical pathway just described, lysosomal damage also induces non-canonical lysophagy. In this process, the ATG12/ATG5/ATG16 complex is recruited to the lysosomal membrane through a V-ATPase-mediated process. Consequently, activated LC3 is directly attached to the lysosomal membrane by non-canonical autophagy and conjugation of ATG8s to single membranes (CASM). This is likely followed by (i) recruitment of the lipid transfer protein ATG2, which is involved in PITT-dependent lysosome repair, and (ii) the fusion of LC3-labeled vesicles with other intact lysosomes [91–93].

### Lysosomal biogenesis and exocytosis

**Fig. 3:**
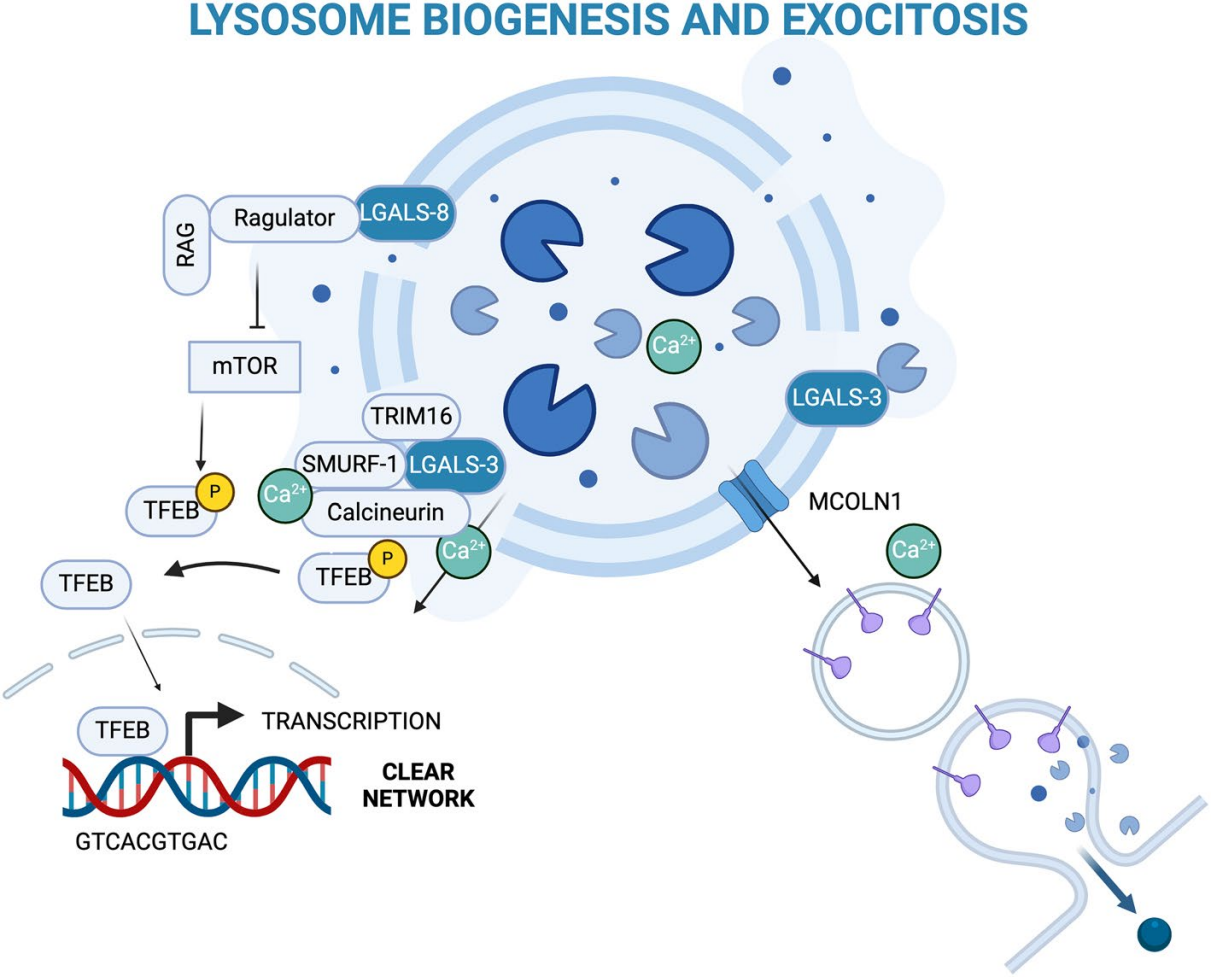
Lysosome biogenesis and exocytosis. To maintain the pool of functional lysosomes the damage of lysosomes activates galectin-dependent mechanisms that induce the transcription factors TFEB and TFE3. Exocytosis of damaged lysosomes with a Ca^2+^-dependent process is induced as an alternative mechanism to lysophagy. Created with BioRender.com

Lysosomal biogenesis and replacement are adaptive mechanisms that maintain the functional pool of lysosomes needed for cellular homeostasis (Fig. 3). These processes depend both on the endocytic pathway and on the biosynthesis of new lysosomal proteins; they further require the coordinated transcription of genes coding for lysosomal and autophagic proteins regulated by TFEB and by its cognate transcription factor E3 (TFE3). These two proteins belong to the microphthalmia (MiT/TFE) family of basic helix-loop-helix-leucin zipper transcription factors, a class of evolutionarily conserved and structurally related proteins. This family includes four members: the microphthalmia transcription factor, TFEB, TFE3, and the transcription factor EC. TFEB and TFE3 recognize a 10 bp palindromic responsive element (GTCACGTGAC), termed the coordinated lysosomal expression and regulation, present in genes controlling the integrated expression of networks regulating autophagy and lysosomal biogenesis, exocytosis [94–97].

TFEB and TFE3 continuously shuttle between cytosol and nucleus, and different stimuli can modify the dynamics of this mechanism. When sufficient nutrients are available, the mTOR complex 1 (mTORC1) inhibits TFEB/TFE3 activity through their phosphorylation (at Ser138, Ser142, or Ser211 for TFEB; at Ser321 for TFE3) [98]. Conversely, starvation or cellular stress conditions promote TFEB activation and nuclear translocation with two parallel mechanisms: by switching off mTORC1 activity and by inducing lysosomal calcium efflux via the mucolipin TRP cation channel 1 (MCOLN1), an event that triggers the activation of the calcium-dependent serine/threonine phosphatase calcineurin that dephosphorylates TFEB/TFE3, thus activating them. [94, 99]. The mitogen-activated protein kinase 1 (MAPK1/ERK2) and the protein kinase C type beta are also involved in TFEB phosphorylation and regulation [100].

How TFEB/TFE3 phosphorylation inhibits their function has been elucidated. Specific phosphorylated serine residues allow both the recognition and the binding of the chaperone YWHA/14-3-3 that retains TFEB/TFE3 in the cytosol [101–104] and mediates their nuclear export via exportin1 (known as chromosomal maintenance 1) [105, 106], thus preventing their nuclear localization.

Beyond phosphorylation, other post-translational modifications control TFEB/TFE3 activity, including acetylation, a process regulated by histone deacetylases (HDAC2, HDAC5, HDAC6, HDAC9) and acetyltransferases (ACAT1, ELP3, CREBP). Treatment with pan-HDAC inhibitors, such as suberoylanilide hydroxamic acid or trichostatin, induces TFEB acetylation and accumulation into the nucleus, promoting lysosomal biogenesis and autophagy [107, 108]. Finally, TFEB/TFE3 are also involved in the redox signaling mediated by the KEAP1/NRF2 pathway, suggesting that lysosomal biogenesis might also be sensitive to the intracellular redox state [109].

In the nucleus, both TFEB and TFE3 exert their transcriptional activity through an LLPS-dependent mechanism involving the formation of physiological protein condensates, which also regulate their activity. It has been demonstrated that the inositol polyphosphate multikinase (IPMK) does not influence TFEB phosphorylation or nuclear translocation, but IPMK can associate with TFEB suppressing its LLPS. IPMK knockdown induces the formation of TFEB condensates promoting its transcriptional activity and leading to autophagy induction and lysosomal biogenesis [110, 111].

Lysosomal damage and LMP have been shown to induce TFEB activation and lysosomal biogenesis to replace the pool of damaged lysosomes cleared by lysophagy [112, 113]. Besides the role of galectin-8 described above, other proteins might be involved in the activation of TFEB following LMP. In kidney injury, it has been observed that lysosomal damage triggers the recruitment of LC3 by the activation of ATG conjugation system. LC3 interacts with MCOLN1 leading to calcium efflux, which induces TFEB nuclear translocation through the activation of calcineurin [114]. Interestingly, it has been recently observed that calcineurin is also indirectly regulated by galectin-3: galectin-3 recruits the SMAD specific E3 ubiquitin-protein ligase SMURF1 to damaged lysosomes, which in turn binds and controls calcineurin promoting its phosphatase activity on TFEB [115].

A peculiar activity of TFEB is its involvement in the regulation of lysosome exocytosis; this process requires that lysosomes fuse directly with the plasma membrane to release their content into the extracellular environment. Lysosomal exocytosis was shown to be involved in the restoration or remodeling of the plasma membrane [116–119], as well as in neurite outgrowth processes and axonal myelinization [120–122].

Lysosomal exocytosis is regulated by lysosomal calcium efflux through the MCOLN1 channel. Interestingly, TFEB overexpression stimulates the activation of MCOLN1 and calcium release from lysosomes mediating lysosomal exocytosis. TFEB and MCOLN1 act in a feedback loop, where TFEB triggers MCOLN1 gene transcription, being a TFEB target gene, while MCOLN1 stimulates TFEB activation via calcineurin, as described above [95].

Recent findings suggest lysosomal exocytosis as a mechanism for the secretion of protein aggregates from neurons, contributing to the maintenance of cellular proteostasis when intracellular degradative systems are impaired [123].

### Lysosome reformation

Beside lysosomal biogenesis, cells can provide a new pool of lysosomes via reformation processes. Lysosomes can also originate through the recycling of the autolysosome membrane via a mechanism known as autophagic lysosome reformation (ALR). ALR involves the protrusion of tubules from autolysosomes, giving rise to small vesicles named proto-lysosomes, which subsequently mature into functional lysosomes [124]. The initiation of ALR is dependent on the reactivation of mTOR. The mechanisms governing mTOR reactivation and the subsequent initiation of ALR during autophagy are still mostly unclear. An important process implicated in this context is the increased production of amino acids, which activates mTOR [124]. Moreover, the release of calcium by lysosomes also plays a role in mTOR activation through a calmodulin-dependent mechanism [125].

The remodeling of autolysosome membranes during ALR is finely regulated by the transient and reversible formation of a specific set of membrane-bound phosphoinositides, following a precise spatiotemporal pattern. Notably, PtdIns P2 recruits clathrin to the autolysosome membrane, which, in turn, stimulates membrane budding [126]. Clathrin additionally serves as a membrane platform to facilitate the accumulation of AP-2-PtdIns P2, which, in turn, promotes the recruitment and clustering of the kinesin family member 5B (KIF5B) protein. KIF5B is a kinesin motor protein that binds to autolysosome membranes and microtubule filaments, thereby facilitating the formation of autolysosome membrane tubules. Finally, the mechanism that promotes the final scission of the lysosome is still unclear [127]. Interestingly, it has been recently demonstrated that, in the presence of severe LMP, the ALR machinery is recruited to damaged lysosomes by TBC1 domain family member 15 to regenerate functional lysosomal membranes. This mechanism represents a prompt cellular response to compensate for the reduction of functional lysosomes before the activation of lysosomal biogenesis mediated by TFEB/TFE3 [128].

Alternatively, lysosomes are also regenerated by the endocytic pathway. Transient “kiss and run” interactions between late endosomes and lysosomes occur to deliver the endocytosed cargoes into the lysosomes. These events result in the formation of endolysosomes, hybrid and heterogeneous organelles from which lysosomes are regenerated with an analogous pathway to that occurring in ALR: characterized by tubulation of endolysosomes, scission, and maturation [129–133].

## Lysosomal damage in neurodegeneration

NDs are fatal progressive disorders characterized by the loss of functionality and/or the death of specific subpopulations of neurons controlling cognitive or motor functions. Different pathological mechanisms induce neuronal death, among which alteration in proteostasis is of great relevance. Proteostasis dysfunction coincides with the generation of damaged organelles involved in the protein quality control (PQC), variation in the expression of contributors to PQC, and the formation of protein aggregates which may ultimately lead to cell death through different mechanisms [134–137]. The most common NDs, depicted in Fig. 2, include Parkinson’s disease (PD), Huntington’s disease (HD), amyotrophic lateral sclerosis (ALS), frontotemporal dementia (FTD), and Alzheimer’s disease (AD). Their classification is based on primary clinical features, anatomical distribution of neuronal degeneration, and the main molecular alterations that characterize each of them. Although these NDs differ significantly in etiopathogenesis and clinical aspects, they generally present certain common cellular and molecular alterations such as protein aggregation, impairment of degradative systems, and damage to degradation-related organelles such as lysosomes. Indeed, emerging evidence suggests that lysosomal dysfunction is strictly correlated with the pathogenesis of these diseases, either as a trigger or a consequence of neuronal or microglial cell dysfunction. LMP and leakage of lysosomal contents, including cathepsin B and calcium, have been observed in various NDs [138–140]. As shown in Table 1, lysosomal damage is associated with ND-related mutations in genes encoding proteins directly involved in lysosomal membrane integrity and lysosomal functionality. These include lysosomal transmembrane proteins such as transmembrane protein (TMEM) 106B and ATPase cation transporting 13A2 (ATP13A2) [141, 142]; or proteins implicated in lysosome repair or degradation, such as LRRK2 and VCP, SQSTM1/p62, OPTN, or TBK1 [143–146] (see below for further details). Of note, neurons can uptake extracellular aggregates through endocytosis; depending upon their nature and biophysical properties, these aggregates can induce lysosomal membrane rupture [55]. Proteins implicated in this mechanism can be found in Table 2. The lysosomal toxicity of protein aggregates has been directly demonstrated for various ND-related proteins, including alpha-synuclein (SNCA), β-amyloid (Aβ), tau, and superoxide dismutase 1 [83, 140, 147–149]. Together, these findings show a strong interaction between lysosomal alterations and NDs.

**Table 1:** Genes mutated in NDs that are involved with lysosomes alterations

Gene	Role	ND-related	References
*LRRK2*	-Recruits and phosphorylates RAB proteins to regulate lysosomal repair -Maintains lysosome pH by interacting with vATPase a1 subunit	PD	[160, 224, 225]
*ATP13A2*	P-type ATPase which maintains lysosome pH	PD	[226]
*GBA*	Lysosomal enzyme degrading glycolipids	PD	[227]
*TMEM175*	Ion channel that contributes in maintaining lysosomal pH	PD	[228]
*SCARB2*	Phospholipid receptor regulator of lysosome-cholesterol interaction	PD	[165]
*SQSTM1/p62*	AR that mediates damaged lysosome engulfment in autophagosomes	ALS/FTD	[144, 229]
*UBQLN2*	Interacts with v-ATPase contributing in lysosomal pH	ALS/FTD	[230, 231]
*DCTN1*	Binds damaged lysosome and promotes their addressing to autophagy degradation	ALS/FTD	[232]
*TBK1*	Phosphorylates AR regulating lysosome degradation	ALS/FTD	[233, 234]
*OPTN*	AR that mediates damaged lysosome engulfment in autophagosomes	ALS/FTD/PD	[145, 188]
*VCP*	Mediates degradation of lysosomal membrane proteins promoting lysosome degradation	ALS/FTD	[146, 235]
*TMEM106B*	Lysosomal transmembrane protein, regulates lysosomal morphology, acidification and transport	FTD	[236]
*MFSD8*	Lysosomal transmembrane protein, indirect regulator of lysosomal calcium content and activity	ALS/FTD	[191, 237]
*CTSF*	Lysosomal enzyme degrading proteins	FTD	[193]
*PGRN*	Modulates lysosome enzymes activity	FTD	[238]
*PSEN1*	Transmembrane protein with enzymatic activity degrading proteins	AD	[239]

**Table 2:** Genes mutated in NDs which induce lysosome alterations

Gene	Role in lysosome damage	ND-related	References
*SNCA*	Toxicity mediated by aggregation	PD	[54, 171]
*C9ORF72*	Toxicity mediated by aggregation	ALS/FTD	[240]
*TARDBP*	Toxicity mediated by aggregation and loss of function	ALS/FTD	[195, 196]
*MAPT*	Toxicity mediated by aggregation and loss of function	FTD/ALS	[199, 200]
*FUS*	Toxicity mediated by aggregation	ALS	[202]
*IT15*	Toxicity mediated by aggregation	HD	[241]
*APP*	Toxicity mediated by aggregation	AD	[215]

### Parkinson’s disease

PD is the most common ND, affecting 1% of the population over 65 years old. The clinical manifestations of the disease can vary among individuals. The most recurrent symptoms and signs include bradykinesia, tremors, muscular rigidity, and speech and cognitive impairments [150]. The histopathological hallmark of PD is the presence of Lewy body (LBs) inclusions, which result from intracellular accumulation of SNCA. LBs are associated with the death of dopaminergic neurons present in the substantia nigra [151]. Other hallmarks of PD include a correlation of the disease with lysosome alterations, such as increased galectin-3 plasma levels, which has been proposed as a potential biomarker to monitor PD-related neurodegeneration [152]. Moreover, patients exhibit an overactivation of microglia that leads to an inflammatory response. The activation of microglia is also associated with lysosomal alterations mediated by galectin-3 [153, 154].

Only 10% of PD cases occur in familiar forms, while 90% are sporadic. To date, a large part of the genetic causes of PD has still to be identified. Approximately 5–10% of hereditary PD cases are associated with identified mutations in genes such as *SNCA*, *LRRK2*, and *PRKN* [155, 156]. Conversely, in most cases, PD etiology is multifactorial and involves an interplay between environmental and genetic factors. Genome-wide association studies have identified various risk genes and *loci* linked to PD [157, 158]; several have a strong link to lysosomes (as reviewed in [159]). In particular, *LRRK2* encodes for a protein involved in lysosome repair thanks to its phosphorylation and interaction with RAB29, which also interacts directly with the a1 subunit of the vacuolar-type ATPase H^+^ pump that maintains lysosomal pH [72, 160, 161]. Other relevant genes are *ATP13A2*, encoding for a cation transporter that maintains the proper pH in lysosomes [162]; *GBA*, encoding for glucocerebrosidase, a lysosomal enzyme that converts glucosylceramide and is involved in lysosome activity [163]; *TMEM175*, encoding for a lysosome channel regulator of potassium in lysosomes [164]; and *SCARB2*, encoding for a structural transmembrane lysosomal protein, which acts as a regulator of cholesterol-membrane composition and a receptor of β-glucocerebrosidase, which in turn controls the clearance of SNCA [165–167]. This long list of genes supports the notion that alterations of lysosome function and dynamics may contribute to PD onset and disease progression. Additionally, other elements correlate PD and lysosome disruption, such as the close relationship between lysosomes misfunctioning and SNCA. This is evidenced by several facts: that lysosomes are essential for SNCA degradation [168] and their alterations or the accumulation of lysosomal substrates result in increased SNCA cytoplasmatic levels, triggering the pathological aggregation [169–171]; that other key players in lysosome repair and clearance, such as galectin-3 and TRIM16, have been shown to promote SNCA release and its spreading into the extracellular environment upon lysosomal damage and to promote SNCA conversion into fibrils [54, 152, 153]; and that SNCA aggregation can alter the autophagic-lysosomal pathway either by directly disrupting lysosomal components or by inhibiting trafficking events [147, 168, 172]. Altogether, these findings show a dual interaction between SNCA aggregation and lysosome functionality, underlying a crucial correlation between them.

### Frontotemporal dementia and amyotrophic lateral sclerosis

FTD and ALS are two distinct NDs that display overlapping clinical signs and pathological mechanisms [173]. FTD primarily affects the frontal and temporal lobes of the brain, leading to changes in behavior, personality, and language skills accompanied by a decline in social cognition, emotional regulation, and executive functions [174]. ALS mainly affects motor neurons, responsible for voluntary muscle control, leading to muscle weakness and paralysis, and impairs the ability to speak, swallow, and breathe [175]. Both FTD and ALS present familial (fFTD and fALS) and sporadic (sFTD and sALS) forms. Although FTD and ALS are distinct diseases, they belong to a spectrum of diseases known as FTD/ALS, which highlights their overlapping nature.

FTD and ALS exhibit common molecular pathological features, including the mislocalization and aggregation of TAR DNA-binding protein 43 (TDP-43), a ribonucleotide protein that regulates mRNA metabolism, the accumulation of FTD/ALS-associated mutated proteins in inclusions, and the failure of the PQC system [173, 176, 177]. FTD/ALS are also associated with alterations to the autophagy–lysosomal pathway, detectable in postmortem tissue of FTD/ALS patients [87, 178] and evidenced by increased levels of galectin-3 in the spinal cord and cerebrospinal fluid, suggesting changes in lysosome dynamics [178, 179].

FTD and ALS also overlap at the genetic level; roughly 30% of fFTD cases, 5–10% of sALS cases, and approximately 50% of fALS cases are linked to a mutation in the *C9ORF72* gene [173, 180]. This mutation involves the abnormal expansion of a hexanucleotide sequence (G_4_C_2_) localized in the first *C9ORF72* intron. This mutation triggers three pathological mechanisms, one related to a loss of function due to haploinsufficiency and the other two involving a gain of toxicity. The toxicity may be caused either by the formation of aberrant RNA *foci* in the nucleus or by an unconventional repeat-associated ATG-independent (RAN) translation, which leads to the production of five different dipeptide repeat proteins. C9ORF72, known as a regulator of autophagic flux, plays a crucial role in maintaining the proper functionality of the autophagy-lysosomal pathway [181]. Haploinsufficiency of C9ORF72 causes the impairment of autophagy and lysosome functions, resulting in the accumulation of lysosome-like organelles that precede neurodegeneration, thereby contributing to the pathogenesis of FTD and ALS (reviewed in [182]). These phenotypes are partially caused by a decreased TFEB expression and by its cytoplasmic retention [183].

Other genes associated with FTD and ALS cases, including *SQSTM1/p62*, *UBQLN2*, *DCTN1*, *TBK1*, *OPTN*, and *VCP* [184–189], have been previously described to play a role in autophagy. Moreover, other genes associated exclusively with FTD are implicated in lysosomal trafficking, including *TMEM106B* and the major facilitator superfamily domain containing 8 (*MFSD8*) or in lysosomal activity, such as *Cathepsin F* (*Ctsf*) and granulin precursor (*GRN*) [190–193]. Thus, mutations in genes associated with lysosomal stability, functioning, or degradation underline an important implication of lysosomes and autophagy in pathological neurodegenerative mechanisms.

Lysosomal alterations in FTD/ALS can also be caused by a gain of toxicity associated with an increased toxic aggregation of TDP-43, mutated proteins such as tau, a microtubule-associated protein, or fused in sarcoma (FUS), a protein involved in regulating RNA metabolism. The aggregation of TDP-43 alters a specific autophagic pathway, chaperone-mediated autophagy (CMA), and disrupts lysosome function, which in turn exacerbates TDP-43 toxicity and loss of function [194, 195]. Indeed, TDP-43 aggregation and functional loss are associated with the activation of autophagosome and lysosome biogenesis through the inhibition of mTORC1 and activation of TFEB. Simultaneously, TDP-43 loss of function causes impairment in the fusion of autophagosomes with lysosomes, via an mTORC1-independent mechanism. Consequently, the buildup of AVs contributes to the aggregation of TDP-43 and neurodegeneration [196]. The aggregation of tau also impairs lysosomal functions through various mechanisms. In physiological conditions, tau stabilizes microtubules, facilitating the proper trafficking and maintenance of lysosomes [197, 198]. However, FTD-associated tau mutants are prone to aggregate, leading to hyperphosphorylation, ubiquitination, and destabilization of microtubules [199]. Moreover, tau aggregates also inhibit IST1, a member of the ESCRT complex, block CMA, and impair lysosome function. This results in the formation of enlarged dysfunctional lysosomes and even their rupture [200, 201]. Similarly, to tau, mutated FUS forms protein aggregates. These aggregates may sequester LAMP-1-positive structures, leading to the aberrant accumulation of functional lysosomes around the abnormal FUS aggregates [202].

### Huntington’s disease

HD is characterized by the progressive deterioration of cognitive, motor, and psychiatric functions. As the disease progresses, HD symptoms include involuntary movements (chorea), cognitive decline, psychiatric disturbances, and difficulties with speech and swallowing [203]. HD is an inherited condition caused by an expansion of CAG trinucleotide repeats in the Huntingtin (*HTT*) gene, resulting in the production of an abnormal form of the HTT protein containing an elongated polyglutamine tract. The mutation leads to the accumulation of toxic protein aggregates in the brain, particularly in the basal ganglia and cortex (Fig. 2). HD displays several hallmarks of impairments in lysosomal function and dynamics. For instance, galectin-3 levels increase in the brains of HD mice and patients, suggesting alterations to lysosome activity. Galectin-3 levels increase in microglia before the onset of the disease and mediate the initiation of the inflammatory response which contribute to HD pathogenesis [204]. Moreover, an increase in the perinuclear accumulation of lysosomes is visible in HD models and it is normalized upon the overexpression of wild-type HTT. Mutant HTT (mHTT)-induced lysosome accumulation is associated with an increase in mTORC1 basal activity and the autophagic flux, resulting in a premature fusion of lysosomes with autophagosomes [205]. To further emphasize the autophagy–lysosome connection with HD, mHTT is recruited to vesicle-rich organelles that resemble multivesicular bodies or autolysosomes, suggesting a lysosome-dependent degradation of mHTT [206]. In addition, lysosomes are implicated in mHTT removal through an unconventional lysosome-dependent secretion mechanism [207].

These findings underscore the importance of an autophagy-lysosome role in HD and provide insights into potential therapeutic targets for the disease.

### Alzheimer’s disease

AD is the most common cause of dementia and primarily affects memory, cognitive abilities, and behavior, gradually impairing daily functioning. AD typically starts with mild memory loss and progresses to severe cognitive decline and loss of independence [208]. The exact cause of AD is not fully understood, but age, genetic factors (such as the apolipoprotein E ε4 allele), and certain lifestyle and environmental factors are believed to play a role in AD pathogenesis [209]. AD is characterized by the accumulation of abnormal protein aggregates in the brain, such as Aβ plaques and tau tangles. Like in the previously discussed NDs, AD also presents signs of altered autophagy–lysosome pathways. This evidence includes galectin-3 accumulation in Aβ plaques in microglia, mediating the maladaptive activation of the inflammatory response [210]; dysregulation in endosome– and lysosome–ER contact sites due to amyloid precursor protein (APP) [211]; increased alkalinization in neuronal lysosomes which appears before Aβ deposition outside the cells. Lysosomal pH alteration is caused by decreased v-ATPase activity, associated with presenilin-1 mutations, and the accumulation of Aβ within enlarged autolysosomes that have lost their acidity. In line with this, in vitro studies have shown that the reacidification of lysosomes rescues lysosome dysfunction and accumulation [212]. Moreover, in affected neurons, AVs containing Aβ accumulate in a tightly packed manner within large membrane protrusions [213–215]. Similar observations have been described in the brains of AD patients. Additional AVs merge to form networks of membrane tubules surrounding the nucleus, where fibrillar Aβ accumulates within the lumens. This leads to the disruption of lysosomal membranes, the release of cathepsins, and ultimately cell death, accompanied by the invasion of microglial cells [215]. Recently it was found that positive modulation of TRIM16-mediated lysophagy decreases the accumulation of Aβ/tau, further underlying the tight connection between lysosome alterations and AD pathology [216].

## Conclusions

Lysosomes are essential organelles for cell viability and alterations in their function are associated with several diseases, including LSDs and NDs. Indeed, accumulating evidence suggests that maintaining lysosomal integrity and efficient lysosomal degradation processes is crucial for neuronal protection and the prevention of NDs. To maintain homeostasis, cells activate different complex mechanisms to repair damaged lysosomes or, when this is not possible, to clear them away through lysophagy or exocytosis. These crucial processes are finely regulated by various proteins and complexes. However, many aspects of these processes remain unknown or not fully understood. Thus, unraveling the complex interplay between lysosomal dysfunction, aggregates accumulation, inflammation, and neuronal cells death holds promise for identifying novel therapeutic targets and developing strategies to counteract or slow down ND progression.

Some steps in this direction have already been taken, and therapeutic approaches and molecules that facilitate lysosomal clearance or biogenesis have been identified. Notably, certain molecules activate lysosome biogenesis by promoting the nuclear localization of TFEB. For example, compounds such as PP 242 and LY 294002 activate TFEB by inhibiting mTOR [217, 218]. Other substances, such as trehalose and its analogs lactulose and melibiose, enhance TFEB activity through an mTOR-independent pathway, as described in ref. [219]. The use of these compounds in disease models has shown promising results, encouraging research in this direction [220–223].

Altogether, this review highlights the intricate nature of the mechanisms governing lysosomal function and dynamics, as well as the consequence of their dysfunction in the development of pathological conditions. The complexity and significance of the described mechanisms underline the necessity of further investigation to enhance our understanding of pathological processes and development of therapeutic strategies.

## Data Availability

Not applicable.

## Abbreviations

AD: Alzheimer’s disease
Aβ: amyloid β
ALR: Autophagic lysosome reformation
ALS: Amyotrophic lateral sclerosis
AMPK: AMP-activated Protein Kinase
APP: Amyloid precursor protein
AR: Autophagy receptor
ATG: Autophagy-related
ATP13A2: ATPase cation transporting 13A2
AV: Autophagic vacuole
CALCOCO2/NDP52: Calcium-binding and coiled-coil domain 2 protein
CASM: Conjugation of ATG8s to single membranes
CHMP4B: Charged multivesicular body protein 4B
CLEAR: Coordinated lysosomal expression and regulation
CMA: Chaperone-mediated autophagy
ESCRT: Endosomal sorting complex required for transport
ER: Endoplasmic reticulum
FTD: Frontotemporal dementia
FUS: Fused in sarcoma
GBA: Glucocerebrosidase
GRN: Granulin precursor
HD: Huntington’s disease
HDAC: Histone deacetylases
HSPA8: Heat shock protein family A (Hsp70) member 8
HTT: Huntingtin
ILVs: Intraluminal vesicles
IPMK: Inositol polyphosphate multikinase
KIF5B: Kinesin family member 5B
LAMP1: Lysosomal associated membrane protein 1
LAMP2A: Lysosome-associated membrane protein 2A
LB: Lewy body
LGALS3: Galectin 3
LIMP: Lysosomal integral membrane proteins
LLPS: Liquid–liquid phase separation
LMP: Lysosomal membrane permeabilization
LQC: Lysosomal quality control
LRRK2: Leucine-rich repeat kinase 2
LSDs: Lysosomal storage disorders
LYTL: Lysosomal tubulation sorting
MAP1LC3: Microtubule Associated Protein 1 Light Chain 3 Beta
MAP3K7/TAK1: Mitogen-activated protein kinase kinase kinase 7
MAPK1: Mitogen-activated protein kinase 1
MCOLN1: Mucolipin TRP cation channel 1
MCS: Membrane contact sites
MFSD8: Major facilitator superfamily domain containing 8
mTOR: Mechanistic target of rapamycin
NDs: Neurodegenerative diseases
OPTN: Optineurin
OSBP: Oxysterol binding protein
PD: Parkinson’s disease
PDCD6IP/ALIX: Programmed cell death 6 interacting protein
PI: Phosphatidyl-inositol
PI4K2A: Phosphatidyl-inositol-4 kinase type 2 alpha
PI4P: Phosphatidyl-inositol 4-phosphate
PITT: Phosphoinositide-initiated membrane tethering and lipid transport
PPP3CB: Protein phosphatase 3 catalytic subunit beta
PQC: Protein quality control
PS: Phosphatidyl-serine
RAN: Repeat-associated ATG independent
SMURF1: SMAD specific E3 ubiquitin-protein ligase
SNCA: Alpha-synuclein
SQSTM1/p62: Sequestosome 1
TARDBP/TDP-43: TAR DNA binding protein
TBK1: TANK-binding kinase 1
TFE3: Transcription factor E3
TFEB: Transcription factor EB
TMEM: Transmembrane protein
TRIM16: Tripartite motif-containing 16
UBA: Ubiquitin-associated
USP9X: Ubiquitin-specific peptidase 9 X linked
VCP: Valosin containing protein
